# Activated Protein C Resistance Does Not Increase Risk for Recurrent Stroke or Death in Stroke Patients

**DOI:** 10.1371/journal.pone.0160382

**Published:** 2016-08-10

**Authors:** Markus Alexander Thaler, Regina Feurer, Christoph Thaler, Natalie Sonntag, Michael Schleef, Ina-Christine Rondak, Holger Poppert

**Affiliations:** 1 Institut für Klinische Chemie und Pathobiochemie, Klinikum rechts der Isar der Technischen Universität München, München, Germany; 2 Neurologische Klinik und Poliklinik, Klinikum rechts der Isar der Technischen Universität München, München, Germany; 3 Institut für Medizinische Statistik und Epidemiologie, Klinikum rechts der Isar der Technischen Universität München, München, Germany; Universita degli Studi di Napoli Federico II, ITALY

## Abstract

**Background:**

Activated protein C (APC) resistance is the most common inherited prothrombotic disorder. The role of APC resistance in ischemic stroke is controversially discussed.

**Objectives:**

The aim of this single center follow up study was to investigate the effect of APC resistance on stroke recurrence and survival in stroke patients.

**Patients/Methods:**

We retrospectively identified 966 patients who had had an ischemic stroke or transitory ischemic attack (TIA) and in whom laboratory tests for APC resistance had been conducted. These patients were contacted to determine the primary outcomes of recurrent ischemic stroke or death.

**Results:**

A total of 858 patients with an average follow up time of 8.48 years were included. APC resistance did not influence cumulative incidence functions for stroke free and total survival. In multivariate analyses, crude and adjusted hazard ratios for recurrent stroke as well as for death where not significantly increased in patients with APC resistance. This also applies to the subgroups of young patients, patients with cryptogenic stroke and patients with atrial fibrillation.

**Conclusion:**

APC-resistance is not a risk factor for subsequent stroke or death in patients with a first ischemic stroke or TIA. Testing for APC-resistance in stroke patients therefore cannot be routinely recommended.

## Introduction

Activated protein C (APC) resistance was first shown to be a possible cause for thrombophilia approximately 20 years ago. Dahlbäck et al. observed that addition of APC to plasma of patients with multiple thrombotic events did not result in the expected prolongation of the coagulation time in an activated partial thromboplastin time (aPTT) assay [1]. In the vast majority of cases, APC resistance is caused by substitution of adenine for guanine at nucleotide 1691 in the factor V (FV) gene [2], a point mutation referred to as FV Leiden. Currently, FV Leiden is assumed to be the most common inherited prothrombotic disorder with the highest carrier frequency of 5.2% in populations of European descent [3].

A distinct association of APC resistance and increased risk for an initial and recurrent venous thromboembolic events was reported early after the disorders first description and is well established [4–6]. The role of APC resistance in arterial infarction, however, is less clear. With respect to stroke, studies yield conflicting results: some found individuals with APC resistance to be more likely to suffer from an ischemic stroke [7–10], others were not able to demonstrate any association [4, 11–30], and yet others only found associations in certain subgroups such as young patients or patients who had a cryptogenic stroke [31–35]. Cryptogenic strokes are strokes with no identifiable cause which account for approximately 30–40% of ischemic strokes [36]. Indications, benefits and drawbacks of testing for inherited thrombophilias within the diagnostic work-up of ischemic stroke patients are therefore controversially discussed [37–41]. Nevertheless, current clinical practice frequently includes testing for FV Leiden in patients with a history of stroke [42], particularly in young patients who have suffered from a cryptogenic stroke.

Deciphering the relationship of APC resistance and stroke is challenging due to the fact, that both conditions are rather common and that stroke is a multifactorial disease with a multitude of possible causes. Concomitant occurrence of APC resistance and stroke is therefore to be expected. Sorting out, if this observation is causal, correlative owing to a primary cause or coincidental is a complex undertaking. Beyond these inherent and hence unchangeable prerequisites, studies performed on APC resistance and stroke generally suffer from two main weaknesses. First, studies have largely been of classical, cross-sectional design limiting significance and impeding correct inferences. Adequate longitudinal or prospective works are lacking in this area of medical research. Additionally, studies to date only insufficiently reflect the actual clinical problem. Whether patients with APC resistance are subjected to higher risks for ischemic strokes is without doubt a question of great theoretical interest. The clinician however is much more interested in the impact of APC resistance on prognosis of stroke patients as this might affect further diagnostic procedures or even therapy.

The effect of APC resistance on stroke recurrence and survival in stroke patients was investigated. To overcome the previously mentioned limitations, we performed a longitudinal, observational follow-up study in which only patients with suspected or actual cerebral ischemia were included.

## Patients/Methods

### Patients

966 consecutive patients admitted to our hospital between April 1995 and June 2006 for suspected or actual cerebral ischemia and for whom a complete diagnostic work-up was available, were considered for the study. All patients had undergone a thorough neurological examination. Electrocardiography (ECG), a detailed sonography of the extra- and intracranial arteries, a 24 h ECG as well as cerebral computed tomography or magnetic resonance imaging or both had been performed. Additionally blood for thrombophilia screening including laboratory tests for APC-resistance had been drawn. Baseline ischaemic events were classified according to slightly modified TOAST criteria by a single physician (RF) blinded for the results of APC resistance testing. The established TOAST criteria [43] were modified in the following way: strokes with conflicting mechanisms were classified as “other etiology” instead of “cryptogenic”, the latter subgroup thereby truly representing strokes without any identifiable cause.

### Laboratory testing for APC-resistance

Functional clotting tests in citrated plasmas were used to screen for APC resistance. For this purpose, the ProC APC reagent from Behring (Marburg, Germany) and FV deficient plasma (FVdp) from Dade (Unterschleißheim, Germany) were used with the Fibrintimer (Behring) until March 1999 (5 μl patient plasma + 45 μl barbital buffer + 50 μl FVdp + 100 μl aPTT reagent, 3 min incubation, + 100 μl CaCl_2_ ± APC). From April 1999 on, the Coatest APC reagent from Chromogenix (Milano, Italy) was used with FVdP on the BCS device (both from Dade Behring, Marburg, Germany) (25 μl 1:10 diluted patient plasma in barbital buffer + 25 μl FVdp + 50 μl aPTT reagent, 3 min incubation, + 50 μl CaCl_2_ ± APC).

Starting in August 1996, DNA was extracted from citrate samples exhibiting abnormal APC-ratios by use of the QIAamp DNA Blood Mini Kit from Qiagen (Hilden, Germany) and FV-Leiden mutation was confirmed by polymerase chain reaction (PCR). Initially, amplification was performed on the Thermocycler GeneAmp PCR System 9700 from Perkin Elmer (Rodgau, Germany) with 5’-CAG AGC AGT TCA ACC AGG-3’ and 5’-CTG AAA GGT TAC TTC AAG GAC-3’ (both from Gibco life technologies, Darmstadt, Germany) as forward and backward primers, respectively. DNA was then fragmented with Mnl I from New England Biolabs (Frankfurt am Main, Germany) and separated in a 2.5% agarose gel electrophoresis. From January 2000 and January 2006 on, confirmation of FV-Leiden however was performed via PCR and fluorescence resonance energy transfer on the Light Cycler 1.5 and Light Cycler 2.0 (both from Roche, Mannheim, Germany), respectively. Extracted DNA was mixed with the LightCycler Red 640-labeled forward primer 5’-TAA TCT GTA AGA GCA GAX TCC-3’ (X = label), the unlabeled backward primer 5’-TGT TAT CAC ACT GGT GCT AA-3’ and the fluorescein-labeled wild type probe 5’-AAT ACC TGT ATT CCT CGC CTG TCX-3’ (all oligonucleotides from Tib Molbiol, Berlin, Germany). After amplification melting curves were recorded from 45°C to 70°C with 0.1°C/s. The wild type allele exhibited a melting curve maximum at 64°C whereas the mutated allele had a maximum at 56°C.

### Collection of follow-up data

Patients being considered were first compared to the follow-up data from a previous study [44] as study cohorts partly overlap. For those known to have died, follow-up data from [44] were used. All “new” patients were initially contacted via mail. If a response was not received, we tried to call the patient directly or–if not successful–the patients relatives. If this also failed, the family doctor was contacted by mail and in absence of an answer by telephone. The last step to establish communication was to contact the population register. In case a new address was available, the patient was again contacted by mail. If we did not receive a reply within two months or in case the population register confirmed the known address, the patient was classified as alive but lost to follow-up for stroke recurrence. In case the population register reported the death of a patient, the respective date was documented and the patient was classified as dead and lost to follow-up for stroke recurrence. Finally, in case the patient could not be found in the population register, he or she was classified as lost to follow-up with respect to survival and stroke recurrence.

A total of 966 patients had initially been identified as eligible for the study. During collection of follow-up data, 20 patients refused to participate and 18 turned out to have moved to an unknown address and therefore dropped out of the study. Hence, the final study cohort consisted of 928 patients out of which follow-up data were available for 858, whereas 70 were lost to follow-up.

Due to the retrospective character of the study a written informed consent could not be obtained in all patients for methodological reasons. Patient information was anonymized and de-identified prior to analysis. The study was approved by the ethics committee of the faculty of medicine of the Technische Universität München (project number: 5731/13).

No financial compensation was provided. Disclosure from the population register is permitted by German law.

### Statistics

Baseline data were compared with the Mann–Whitney U test for quantitative variables and with the χ^2^ test or Fisher’s exact test for qualitative variables as applicable.

Recurrent stroke and death were considered as competing events and the Aalen-Johansen [45] estimator was used to compute cumulative incidence functions (i.e. the cumulative probability for a certain event up to a given time) for both APC risk groups. Log-rank tests were used to compare cause-specific hazard rates between APC risk groups. The effect of APC and prognostic factors on these cause-specific hazard rates was quantified using univariate and multivariable Cox proportional-hazard models while the occurrence of a competing endpoint or lost to follow-up was defined as censored observation [46, 47]. Only prognostic factors that showed a statistically significant effect were included in multivariable model.

Regression models were also computed in the following patient subgroups: age < 50 years, initial cryptogenic stroke and atrial fibrillation.

All reported P values are two-sided, with a significance level of 0.05 and have not been adjusted for multiple testing.

Statistical analyses were performed with SPSS version 21 from IBM (Ehningen, Germany) and R version 3.0.0 [48] using the packages “cmprsk” [49] and “mstate” [46, 50, 51].

## Results

### Baseline data of included and excluded patients

The study cohort comprised a total of 928 patients out of which for 858 (92.5%) follow-up data were available. Average follow up time for participants of the study was 8.48 years, ranging from 0.03 to 17.67 years.

Baseline data of patients included as well as of the subjects lost to follow-up and comparison are given in Table 1. Participants included in the study were significantly older (in median 59 vs. 51 years; p < 0.001) and more likely to suffer from hypertension (59.3% vs. 47.1%; p = 0.047) than those lost to follow-up. With respect to the other baseline data, no significant differences between included patients and patients lost to follow-up could be demonstrated.

**Table 1:** Baseline data of considered patients.

parameter	Included	lost to follow-up	p
number	858 (100.0%)	70 (100.0%)	-
male	540 (62.9%)	37 (52.9%)	^‡^0.094
median age [years] (range) [years]	59 (14–91)	51 (20–79)	^†^<0.001
TOAST 1	98 (11.4%)	7 (10.0%)	^‡^0.301
TOAST 2	197 (23.0%)	12 (17.1%)	
TOAST 3	184 (21.4%)	12 (17.1%)	
TOAST 4	59 (6.9%)	3 (4.3%)	
TOAST 5 (cryptogenic)	315 (36.7%)	35 (50.0%)	
TOAST 6	5 (0.6%)	1 (1.4%)	
APC resistance	72 (8.4%)	4 (5.7%)	^‡^0.432
heterozygous	68 (7.9%)	4 (5.7%)	^*^0.741
homozygous	4 (0.5%)	0 (0.0%)	
hypertension	509 (59.3%)	33 (47.1%)	^‡^0.047
diabetes mellitus	140 (16.3%)	6 (8.6%)	^‡^0.087
smoking	362 (42.2%)	32 (45.7%)	^‡^0.566
hyperlipoproteinaemia	340 (39.6%)	27 (38.6%)	^‡^0.862

Baseline data of included patients and of patients lost to follow-up.

* Fisher’s exact test

† Mann–Whitney U test

‡ χ²-test

### Baseline data of patients with and without APC resistance

The study cohort was subdivided into two groups according to the presence or absence of APC resistance. Comparison of baseline data shown in Table 2 did not reveal any significant differences.

**Table 2:** Baseline data of included patients.

parameter	no APC resistance	APC resistance	p
number	786 (100.0%)	72 (100.0%)	-
Male	492 (62.6%)	48 (66.7%)	^‡^0.494
median age [years] (range) [years]	60 (14–87)	56 (27–91)	^†^0.514
hypertension	461 (58.7%)	48 (66.7%)	^‡^0.185
diabetes mellitus	128 (16.3%)	12 (16.7%)	^‡^0.933
smoking	333 (42.4%)	29 (40.3%)	^‡^0.731
hyperlipoproteinaemia	313(39.8%)	27 (37.5%)	^‡^0.700
history of myocardial infarction	48 (6.1%)	7 (9.7%)	^*^0.213
atrial fibrillation	101 (12.8%)	7 (9.7%)	^‡^0.444
TOAST 5 (cryptogenic)	290 (36.9%)	25 (34.7%)	^‡^0.714

Baseline data of patients with and without APC resistance.

* Fisher’s exact test

† Mann–Whitney U test

‡ χ²-test

### Cumulative incidence functions

**Fig 1:**
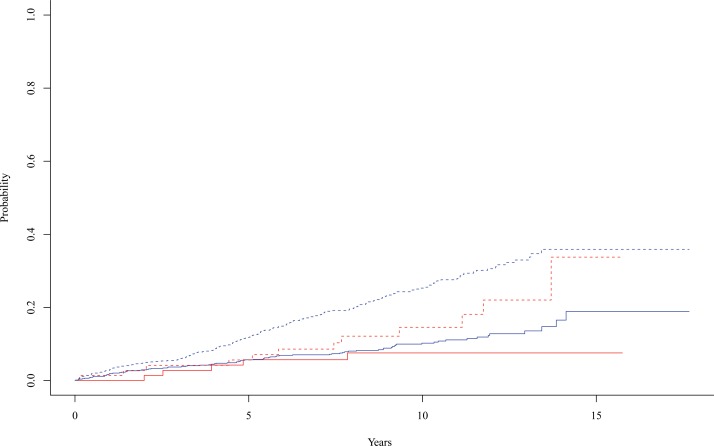
Incidence functions. Cumulative incidence functions for the two competing endpoints “stroke first” (solid line) and “death first” (dotted line) according to the presence (red) or absence (blue) of APC resistance.

The endpoint “stroke first” was reached by 84 and 5 and the endpoint “death first” by 193 and 12 patients for individuals without and with APC resistance, respectively. Cumulative incidence functions for the two competing endpoints according to the presence or absence of APC resistance are depicted in Fig 1. After 10 years, 7.6% of the patients with and 10.2% of those without APC resistance had had a recurrent event. As well after 10 years, 14.6% and 25.3% had died without experiencing a recurrent stroke in the groups with and without APC resistance, respectively. Stroke free survival (endpoint “stroke first”) as well as total survival for patients not suffering from a recurrent stroke (endpoint “death first”) did not differ significantly between patients with and without APC resistance (log-rank test; p = 0.3187 and p = 0.0761, respectively).

### Cox regression

In univariate analysis, only history of myocardial infarction was associated with a significantly increased HR regarding the endpoint “stroke first”. With respect to the endpoint “death first” however age, hypertension, diabetes mellitus and again history of myocardial infarction were associated with a significantly increased in contrast to female sex and cryptogenic stroke with a significantly decreased HR (see Table 3). Hence, only the aforementioned variables were considered for the subsequent multivariate analyses.

**Table 3:** Univariate analysis.

	stroke first			death first		
parameter	HR	95% CI	p	HR	95% CI	p
sex	0.899	0.583–1.386	0.629	0.737	0.603–0.863^*^	<0.001^*^
age	1.014	0.999–1.030	0.063	1.078	1.064–1.092^*^	<0.001^*^
hypertension	1.455	0.942–2.247	0.091	2.857	2.056–3.970^*^	<0.001^*^
diabetes mellitus	1.496	0.880–2.544	0.137	2.849	2.114–3.839^*^	<0.001^*^
smoking	1.072	0.704–1.632	0.747	1.237	0.940–1.628	0.129
hyperlipoprotein-aemia	1.249	0.821–1.900	0.299	1.001	0.755–1.328	0.992
history of myocardial infarction	2.102	1.054–4.193^*^	0.035^*^	2.680	1.762–4.076^*^	<0.001^*^
atrial fibrillation	1.080	0.588–1.985	0.804	1.407	0.978–2.025	0.066
TOAST 5 (cryptogenic)	0.718	0.459–1.124	0.147	0.555	0.407–0.757^*^	<0.001^*^

Univariate analysis of the influence of possible confounding factors on the event specific HR of the endpoints “stroke first” and “death first”.

* significant values

Multivariate analyses demonstrate the HR for recurrent stroke as well as for death without recurrent stroke in patients with APC resistance not to be significantly increased as compared to patients without. This observation holds true for crude HR without any adjustments as well as for HR adjusted for age and sex, for age, sex and presence of cryptogenic stroke and for age, sex, presence of cryptogenic stroke and cardiovascular risk factors (Table 4).

**Table 4:** Multivariate analysis for all included patients.

endpoint	adjusted for	HR	95% CI	p
stroke first	-	0.568	0.230–1.402	0.220
	age & sex	0.574	0.233–1.417	0.229
	age & sex & cryptogenic stroke	0.572	0.232–1.412	0.226
	age & sex & cryptogenic stroke & cardiovascular risk factors*	0.546	0.221–1.348	0.189
death first	-	0.592	0.330–1.060	0.078
	age & sex	0.701	0.390–1.259	0.234
	age & sex & cryptogenic stroke	0.696	0.388–1.251	0.226
	age & sex & cryptogenic stroke & cardiovascular risk factors*	0.613	0.340–1.103	0.103

Cox proportional hazard model: subjects with vs. subjects without APC resistance for all included patients.

*hypertension, diabetes mellitus, history of myocardial infarction

APC resistance is speculated to play a critical role in young stroke patients, in cryptogenic stroke and in patients with atrial fibrillation. The aforementioned multivariate analyses where therefore repeated separately for each of these three subgroups (Tables 5–7). Likewise, presence of APC resistance did not result in a significantly increased HR for any of the two endpoints “stroke first” or “death first”, irrespective of the investigated subgroup and of adjustments for different variables.

**Table 5:** Multivariate analysis for young patients.

endpoint	adjusted for	HR	95% CI	p
stroke first	-	0.043	0.000–53.720	0.387
death first	-	1.825	0.404–8.245	0.434
	age & sex	1.579	0.338–7.385	0.561
	age & sex & cryptogenic stroke	1.509	0.325–6.97	0.599
	age & sex & cryptogenic stroke & cardiovascular risk factors*	1.853	0.309–8.795	0.438

Cox proportional hazard model: subjects with vs. subjects without APC resistance for patients < 50 years (n = 232). Due to numerical reasons, no adjusted models were fitted for the endpoint “stroke first”.

*hypertension, diabetes mellitus, history of myocardial infarction

**Table 6:** Multivariate analysis for cryptogenic stroke patients.

endpoint	adjusted for	HR	95% CI	p
stroke first	-	0.333	0.045–2.464	0.281
	age & sex	0.336	0.045–2.495	0.287
	age & sex & cardiovascular risk factors*	0.355	0.048–2.647	0.313
death first	-	0.601	0.187–1.928	0.392
	age & sex	0.766	0.237–2.481	0.657
	age & sex & cardiovascular risk factors*	0.724	0.233–2.348	0.590

Cox proportional hazard model: subjects with vs. subjects without APC resistance for patients with cryptogenic stroke (n = 315).

*hypertension, diabetes mellitus, history of myocardial infarction

**Table 7:** Multivariate analysis for atrial fibrillation patients.

endpoint	adjusted for	HR	95% CI	p
stroke first	-	1.115	0.141–8.796	0.918
	age & sex	0.851	0.102–7.100	0.882
	age & sex & cryptogenic stroke	0.805	0.096–6.726	0.841
	age & sex & cryptogenic stroke & cardiovascular risk factors*	0.963	0.112–8.286	0.972
death first	-	1.079	0.329–3.542	0.900
	age & sex	1.159	0.347–3.877	0.810
	age & sex & cryptogenic stroke	1.188	0.354–3.987	0.780
	age & sex & cryptogenic stroke & cardiovascular risk factors*	1.347	0.390–4.654	0.638

Cox proportional hazard model: subjects with vs. subjects without APC resistance for patients with atrial fibrillation (n = 108).

*hypertension, diabetes mellitus, history of myocardial infarction

## Discussion

We investigated a cohort of unselected, adult patients admitted to hospital for suspected or actual cerebral ischemia. The data gathered suggest, that presence or absence of APC-resistance does not influence risk for a subsequent stroke episode or death. This finding also applies to the young, cryptogenic stroke and atrial fibrillation patient subgroups.

Since the discovery of APC-resistance, association with arterial ischemic events and in particular stroke has been investigated intensively. All of these studies employed a cross-sectional setup. Yet, to the best of our knowledge, the presented study is the only one of longitudinal and observational design on APC-resistance and stroke.

Previously published studies report in part conflicting results as compared to the presented work. Amongst them, some demonstrated that in consecutive stroke patients prevalence of APC-resistance is generally higher than in controls [7–10]. Others failed to show a significant association within the total, unselected study cohort and in this regard were comparable to our study. They were, however, contrary to the presented work, able to establish a correlation in defined subgroups such as young patients with cryptogenic stroke [31], women using oral contraceptives [32], young female smokers [33] or patients exhibiting several prothrombotic genetic risk factors [34, 35].

The vast majority of the aforementioned studies included less than half of the subjects enrolled in our study and all of them were cross-sectional analyses–both factors impairing the studies statistical relevance. Two of the studies finding an association in the total study cohort investigated individuals with ethnicities different from the population of European descent studied in the presented work [9, 10]. APC-resistance may be caused more frequently by mutations other than FV Leiden in these populations [9]. Finally, different risk factors for stroke exhibit varying risks for recurrence (e. g. high for arterio-arterial etiologies and for cardioembolism due to atrial fibrillation, low for cryptogenic strokes). A high prevalence of APC-resistance in stroke patients therefore does not necessarily contradict our finding of a low risk for stroke recurrence. If APC-resistance is a slight risk factor for a first stroke, risk for recurrence might be minimal in this subgroup.

Consistent with our follow-up data, several cross-sectional original studies [4, 11–18] as well as one large-scale meta-analysis [19] examined unselected cohorts of adult stroke patients and found prevalences of APC-resistance comparable to healthy controls. Consequently, as in our study, odds ratios for stroke were not increased by the presence of APC-resistance. Furthermore, a considerable number of studies analysed distinctly defined subgroups of stroke patients. Whereas only one study explicitly investigated the relationship of APC-resistance and stroke in elderly patients [20], extensive research on this topic was conducted in young adult populations [21–27] as a more pronounced effect was hypothesized. None of these aforementioned, cross-sectional studies could demonstrate a significant impact of APC-resistance on stroke risk even when considering only young patients. This finding is consistent with our results when analysing patients younger than 50 years. Finally, a few studies specifically focusing on young adults with cryptogenic stroke again were not able to establish a significant association between APC-resistance and stroke [28–30]. Our results are well in line with these studies as analysis of patients with cryptogenic stroke only provided no evidence for a peculiar role of APC-resistance in this subgroup.

The presented work not only confirms the findings of the majority of studies but also substantiates and extends knowledge on APC-resistance and stroke with regard to several aspects. The follow-up character of the study provides superior data compared to the commonly applied cross-sectional approach. Our study further simulates the actual scenario in patient care, i.e. the question not being if subjects with APC-resistance suffer from strokes more often but if patients with a first stroke episode differ in their prognosis dependent on the presence or absence of APC-resistance. With exception of the meta-analyses, considerably more patients were included than in all other publications. Additionally, the exceptionally long follow-up time of 8.5 years with only 7.9% of patients lost to follow-up add to the quality. Finally, we not only investigated the role of APC-resistance with respect to the endpoint stroke but also with respect to survival as the most rigorous endpoint possible.

Some limitations of the study presented herein have to be mentioned. First, though considering consecutive patients with cerebral ischemia, only those with APC-resistance test results were included—thereby creating a certain preselection. Second, included patients and patients lost to follow-up differed with respect to age and prevalence of hypertension. A possible effect of the incomplete follow-up on the results cannot be excluded. Furthermore, for 210 patients only data on survival were available and not on a possible recurrent stroke. In the chosen statistical approach, those patients were considered to not have suffered from recurrent stroke. The results therefore constitute a best case scenario. We accepted this drawback as alternative approaches would not have allowed taking into account the information included in these 210 patients. Finally, information on stroke recurrence was mainly provided by the patients themselves or relatives. Erroneous data due to limited retrospection and lack of documentation may have occurred.

In conclusion, our data suggest that APC-resistance is not a risk factor for a subsequent stroke or death in patients with a first stroke episode–in the unselected cohort as well as in young patients, in patients with cryptogenic stroke and in patients with atrial fibrillation. Cost effectiveness of thrombophilia screening in patients with ischemic stroke has been generally questioned due to lack of therapeutic consequences [52, 53]. Our results strongly support the opinion, that testing for APC-resistance and Factor V Leiden in stroke patients is dispensable for pure medical reasons. The utility of testing for other thrombophilic risk factors in stroke patients remains a question for further research.

## Supporting Information

S1 File Raw data underlying the findings described. (CSV)

S2 File Description of the raw data. (TXT)

## Abbreviations

APC: activated protein C
aPTT: activated partial thromboplastin time
ECG: Electrocardiography
FV: factor V
FVdp: factor V deficient plasma
HR: hazard ratio
PCR: polymerase chain reaction
TIA: transitory ischemic attack
